# Lensless Scheme for Measuring Laser Aberrations Based on Computer-Generated Holograms

**DOI:** 10.3390/s20154310

**Published:** 2020-08-02

**Authors:** George Krasin, Michael Kovalev, Nikita Stsepuro, Pavel Ruchka, Sergey Odinokov

**Affiliations:** 1 Laser and Optoelectronic Systems Department, Bauman Moscow State Technical University, 2nd Baumanskaya st. 5/1, 105005 Moscow, Russia; m.s.kovalev@gmail.com (M.K.); sng.bmstu.rl@gmail.com (N.S.); odinokov@bmstu.ru (S.O.); 2 Insistute of Functional Matter and Quantum Technologies, University of Stuttgart, Allmandring 3, 70569 Stuttgart, Germany; paul.ruchka@gmail.com

**Keywords:** phase aberrations, wavefront sensor, computer optics, computer-generated hologram, spatial light modulator, diffraction

## Abstract

All of the existing holographic wavefront sensors are either bulky or have low accuracy of measuring wavefront aberrations. In this paper, we present an improvement of the holographic method of measuring wavefront aberrations using computer-generated Fourier holograms. The novelty of this work lies in the proposed approach to the synthesis of Fourier holograms, which are implemented using phase-only SLM. The main advantages of this method are the increased diffraction efficiency compared to the previously known methods, and the more compact implementation scheme due to the elimination of the conventional Fourier-lens. The efficiency of the proposed method was confirmed by numerical simulation and optical experiments.

## 1. Introduction

New methods and devices with a wide variety of properties and parameters [1,2,3] are being developed based on controllable optical components due to the variety and flexibility of such devices. Microdisplays are used more and more often as metrological tools for monitoring parameters of laser beams, in adaptive atmospheric sounding systems, imaging optical holography systems, etc. [4,5]. The development of computer technologies as well as solid-state singe pixel and matrix optical detectors has created a new direction in holography—Digital holography. This direction is based on the registration of a hologram using a matrix detector and subsequent reconstruction of the objective wave using computer algorithms, which in turn has become a common tool for research and practical applications in various fields. Another direction that differs from digital holography is computer holography. It includes such methods as numerical calculation of an interference pattern from the object and reference waves (hologram), methods of mathematical reconstruction and transformation of generated wave fields, as well as optical reconstruction of the resulting computer-generated hologram using the spatial light modulator. Digital and computer holography methods provide more opportunities for analyzing the amplitude and phase of an optical field in comparison with the traditional optical process and allow for the implementation of a holographic wavefront sensor, which provides additional method flexibility [6,7].

There are various methods for measuring laser beam aberrations: interferometric methods, Hartmann method, and holographic methods, but the most used is the implementation of the Hartmann method—the Shack–Hartmann sensor [8,9,10]. Interferometric methods have the best accuracy among all methods (from λ/40 to λ/1000), but due to their limitations they are only applicable for solving a rather narrow range of problems and cannot be an optimal and general solution for problems of detecting distortions of optical wave fields [11,12,13,14]. The Hartmann method is a fairly simple and universal way of measuring the phase of optical wave fronts, with its subsequent interpretation in the form of distortions [15,16]. However, the need to reconstruct the phase from its spatial derivative makes the Hartmann method often inapplicable in certain problems. Thus, it is necessary to investigate and develop a new method for measuring aberrations of the wavefront of laser beams with increased accuracy and wide dynamic range based on the principles of computer and digital holography, which do not require the creation of physical standards and operate in a modal mode at large apertures.

This paper is aimed at developing a method for measuring laser beam aberrations based on the principles of computer holography, which has high measurement accuracy, a wide range of applications, and a compact device for its implementation.

## 2. Materials and Methods

### 2.1. Algorithm for Measuring Wavefront Aberrations

For the problem of sensing wavefront aberrations, it is common to mathematically represent the phase function of a beam as a serial expansion of orthogonal basis functions such as $\psi \left(x,y\right)=\sum_{n}\alpha_{n}z_{n}\left(x,y\right)$, where $\left\{z_{n}\left(x,\;y\right)\right\}$ is a set of basis functions and $\left\{\alpha_{n}\right\}$ is a set of weight coefficients. There are numerous basis functions representations known nowadays. Zernike polynomials (or modes) are among the most used. However, we must note the main drawback of Zernike polynomials for solving the problem of wavefront aberrations sensing: basis functions are orthogonal only for a circular aperture, whereas they are not orthogonal for any general discrete representation of wavefront [17]. Another successful example of basis functions representation for wavefront description is modified Zernike polynomials for hexagonal, elliptical, and rectangular apertures [18], and for discrete set of points [19]—2D Chebyshev polynomials [20], Karhunen-Loève functions [21,22], etc. Selection of the method depends on the conditions of the problem to be solved. One can use mathematical optimization methods to find the coefficients $\left\{\alpha_{n}\right\}$. The problem can be described as

(1) $$\left\{{\tilde{\alpha }}_{n}\right\}=\underset{\left\{\alpha_{n}\right\}\in R^{N}}{\mathrm{argmax}}J\left(\left\{\alpha_{n}\right\}\right)$$

where $J\left(\left\{\alpha_{n}\right\}\right)$ is optimization function and N is the number of basis functions used in the task.

An output peak amplitude can be used as optimization function. Selection of peak intensity as optimization function allows one to measure only a small fraction of output field and simplify the procedure of $J\left(\left\{\alpha_{n}\right\}\right)$ calculation. However, this criterion is sensitive to fluctuations in the power of the light source. To solve this problem, various normalized correlation metrics such as peak-to-sidelobe ratio (PSR) or peak-correlation energy (PCE) can be used [23]. In previous works of the authors [4,24], it was shown that convex optimization methods can be applied to solve the problem (1) in the range of ±λ/2 of wavefront aberrations, which can be increased up to ±5λ without loss in accuracy. Gradient descent, Newton’s method, or particle swarm are among of the lots of other methods [25], which could be implemented to solve the problem (1).

**Figure 1:**
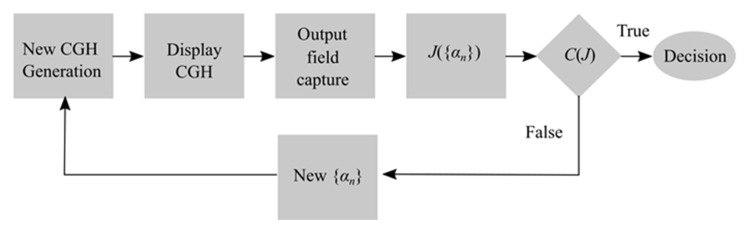
Block diagram of the algorithm for measuring wavefront aberrations.

Figure 1 illustrates the block diagram of wavefront aberrations measuring algorithm. First, a zero-step CGH structure is synthesized using the starting set of $\left\{\alpha_{n}\right\}$, which could be specially predetermined or assigned randomly. This step starts an iteration algorithm, which includes next basic steps:

1. Selection of $\left\{\alpha_{n}\right\}$ and calculation of temporary wavefront model $\psi_{t}\left(x,y\right);$
2. Synthesis of CGH structure;
3. Displaying the CGH structure by SLM;
4. Capturing of output intensity distribution by CCD camera;
5. Searching for correlation peak position and determining the optimization function value;
6. Checking the termination condition of the algorithm. If condition is satisfied, go to step 7, otherwise repeat steps 1–6;
7. Decision.

It should be noted that this algorithm is free from the influence of cross-correlation noise, a major drawback of all holographic wavefront sensors that restricts achievable accuracy, because only one CGH is outputted on the SLM at a time. Hologram itself is generated from the phase function of the wavefront, which can consist of many different aberrations.

### 2.2. Modified Wavefront Aberration Measurement Algorithm

An iterative algorithm was implemented in [4], which allows one to detect any number of aberrations. However, the implementation of this method requires the use of a Fourier transform lens, which imposes certain requirements on the alignment of the system and leads to an increase in its size. Typically, there are standard lenses with a limited number of focal lengths, while lenses with custom focal lengths are usually expensive and hard to come by. At the same time, the use of modern phase SLM, in particular modulators with a large phase dynamic range, allows one to replace this optical element with its digital analogue—a generated Fresnel lens.

The possibility of deriving a Fresnel digital lens along with a Fourier hologram was shown in [3]. This approach allows one to get rid of the Fourier transform lens in the optical setup. Consider the procedure for setting a digital Fresnel lens with focus f.

The phase function of a classical spherical lens can be written as

(2) $$\phi_{l}\left(x,y\right)=-k\cdot \frac{x^{2}+y^{2}}{2f},$$

where k=2π/λ denotes wave number.

The transmittance function of the lens can be written as

(3) $$L_{l}\left(x,y\right)=\exp\left(j\phi_{l}\left(x,y\right)\right).$$

In previous works [4,24], the hologram was based on a sinusoidal grating; therefore, a large number of diffraction maxima were observed in the registration plane. However, their large number in the scheme proposed in this article does not lead to any positive effects. Thus, it was decided to replace the sinusoidal grating with a blazed one, oriented along the Ox axis. Blazed grating concentrates the incident light of the corresponding wavelength mainly in one diffraction maximum.

The reflection coefficient of such grating can be mathematically represented as

(4) l(x,y=0)=exp[jkxsinβ],

where β denotes a reflection angle.

Let the phase function of the wavefront that would be encoded into CGH be equal to

(5) $$\hat{h}\left(x,y\right)=\exp\left[jk\psi \left(x,y\right)\right],$$

where ψ(x,y) is the wavefront function.

The resulting hologram phase function is written as

(6) $$h\left(x,y\right)=\hat{h}\left(x,y\right)l\left(x,y\right)=\exp\left[jk\left(\psi \left(x,y\right)+xsin\beta \right)\right].$$

Then, the combined function of the hologram with the digital Fresnel lens can be represented as

(7) $$h_{l}\left(x,y\right)=\exp\left[jk\left(\psi \left(x,y\right)+x\sin\beta \right)+j\phi_{l}\left(x,y\right)\right].$$

If g(x,y) is the function of the testing beam in the plane of the hologram, then at a distance f from the hologram, the amplitude distribution of a scattered beam can be found using the Fresnel transform from the product $g\left(x,y\right)\cdot h_{l}\left(x,y\right)$

(8) $$A\left(x^{\prime },y^{\prime }\right)=\exp\left[jk\frac{{x^{\prime }}^{2}+{y^{\prime }}^{2}}{2f}\right]ℱ\left\{g\left(x,y\right)h_{l}\left(x,y\right)\exp\left[jk\frac{x^{2}+y^{2}}{2f}\right]\right\}\left[\frac{x^{\prime }}{2f},\frac{y^{\prime }}{2f}\right],$$

where $ℱ\left\{f\left(x,\;y\right)\right\}\left[x^{\prime },y^{\prime }\right]$ is the symbol of the Fourier transform of the function f(x,y) with respect to spatial frequencies $\left[x^{\prime },y^{\prime }\right]$. Substituting (2) and (7) into (8), the amplitude of the light beam in the output plane can be obtained up to a phase factor in the form

(9) $$A\left(x^{\prime },y^{\prime }\right)\propto \left[G*H\right]\left(x^{\prime }-f\sin\beta ,\;y^{\prime }\right),$$

where the square brackets show the correlation of the Fourier transforms of the testing beam function g(x,y) and the wavefront phase function $\hat{h}\left(x,y\right)=\exp\left[jk\psi \left(x,y\right)\right]$ encoded in the CGH.

**Figure 2:**
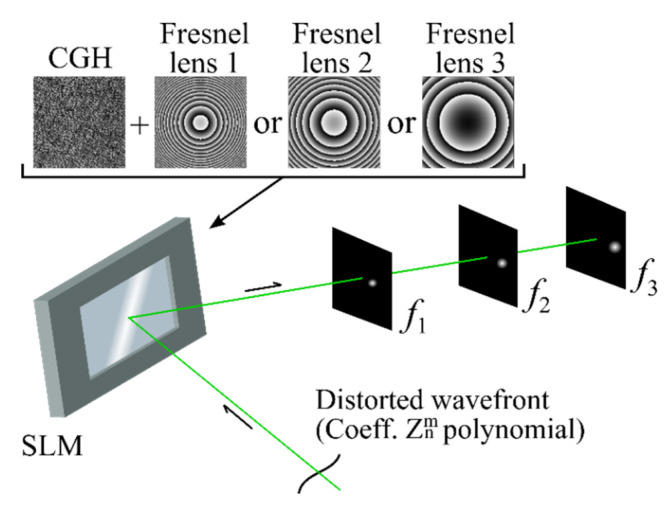
Functional scheme of the modified wavefront measurement algorithm operating principle.

Figure 2 depicts the principle of the modified algorithm operation. The distorted laser beam is incident on the phase SLM, where CGHs with Fresnel lenses are outputted. The distorted beam denotes a beam into which wavefront aberrations were introduced using, for example, an adaptive mirror. Thus, a correlation peak is formed in the registration plane. The size and intensity of the peak depend on the presence and magnitude of the wavefront aberrations in the beam incident on the SLM. When the values of the wavefront aberrations in the incident beam and wavefront aberrations encoded in the GGH coincide, the correlation peak is a delta function and has a minimum size. Figure 2 also shows that the correlation peak will form in different planes, which are determined by the focal length of the digital Fresnel lens.

## 3. Results and Discussion

In the experimental part of the study, a scheme that allows automatic correlation detection of wavefront aberrations was proposed. A phase SLM of reflective type (Holoeye PLUTO-2-VIS-016, 1920 × 1080, pixel size 8 μm) was used to output CGHs. To ensure maximum efficiency of the method, the polarization of the incident on the SLM radiation was oriented horizontally using linear polarizer (Thorlabs LPVISC050-MP2). The spatial orientation of the incident beam does not play a big role, as long as the angle between the incident beam and the SLM differs from the normal by no more than 3–4° [26]. In this case, the phase factor responsible for the slope can be neglected, since it does not affect the shape of the correlation peak.

**Figure 3:**
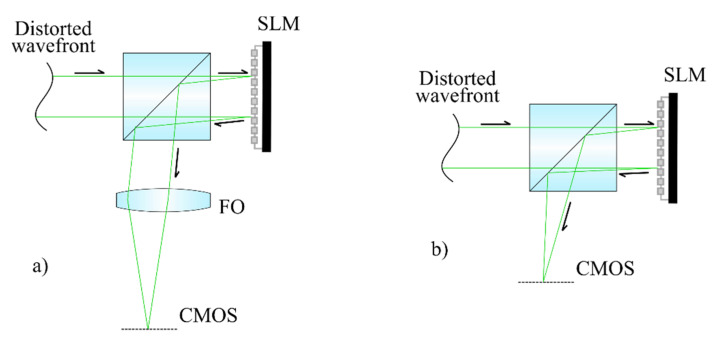
The optical scheme of the wavefront sensor with a Fourier transform lens (**a**) and the proposed scheme with a digital Fresnel lens (**b**).

The scheme of controlled distortion formation is similar to that considered previously [27]. In all experiments, one or more aberrations (with amplitude value of each of which was equal to 1.5λ) were introduced into the laser beam formed by a diode-pumped semiconductor laser (Cobolt 04-51, λ = 660 nm) using a deformable mirror (Thorlabs DMP40/M-P01) in feedback from the Shack–Hartmann wavefront sensor (Thorlabs WFS300-14AR). A monochrome CMOS camera (Thorlabs CS2100M-USB, 1920 × 1080, pixel size 5.04 μm) was used to register the intensity distribution. In this work, a comparison between the proposed method (Figure 3b) and the method previously proposed on the basis of a physical Fourier transform lens [4,24] (Figure 3a) was made. It should be noted that to simulate and measure wavefront aberrations, Zernike polynomials were used. The main reasons for their choice were that deformable mirror and Shack–Hartmann wavefront sensor work only using this basis.

**Figure 4:**
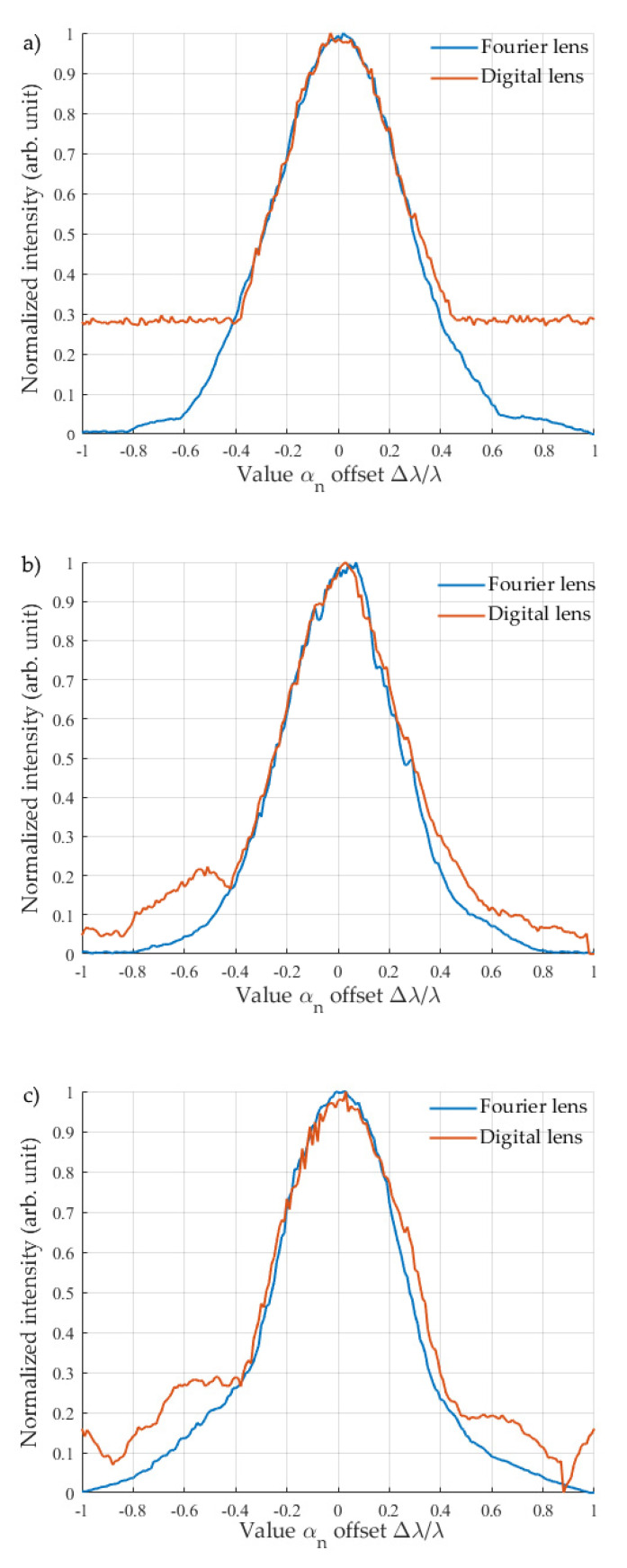
Comparison of the correlation functions obtained via two methods for lenses with a focal length equal to 250 mm (**a**), 500 mm (**b**), and 1000 mm (**c**).

Firstly, correlation functions of a single aberration (vertical astigmatism $Z_{2}^{2}$) were obtained using classical Fourier transform lenses with a focal length of 250, 500, and 1000 mm. Further, using the same scheme, correlation functions for two aberrations (vertical astigmatism $Z_{2}^{2}$ and vertical coma $Z_{3}^{-1}$) were recorded. Finally, the second group of experiments based on digital Fresnel lenses with identical focal lengths demonstrates the applicability of the proposed method for measuring wavefront aberrations. A comparison of the obtained normalized correlation functions during the detection of one aberration is shown in Figure 4. One can see that for the proposed method, the shape of the correlation function, its growth rate, and the location of maximum is identical to the parameters of the correlation function obtained using classical scheme. It should be noted that the absolute values of the maxima of the correlation functions for the method based on digital Fresnel lenses turned out to be less than that when using a traditional Fourier lens, namely, 5.85, 3.17, and 3.67 times less for lenses with focal lengths of 250 mm, 500 mm, and 1000 mm, respectively. Thus, one can say that the overall energy incident on the CMOS decreases, along with the signal-to-noise ratio. However, even with such a signal-to-noise ratio, the absolute value of the maximum of the correlation function is several times higher than the noise, which is sufficient to determine the wavefront aberrations without loss of accuracy.

The side lobes on the graphs can be explained by the features of the algorithm. Local maxima will appear when the value of the wave aberration in the CGH is a multiple of but not equal to the value thereof in the tested beam. This was described in more detail in [24]. The side lobes presence or absence on graphs is also determined by the signal-to-noise ratio. As mentioned above, the noise level for the case of digital Fresnel lens with a focal length of 250 mm was two times higher than that for other cases. Thus, the side lobes were “drowned” in noise.

Table 1 shows the values of the correlation function peaks for the case of detecting two wavefront aberrations. As can be seen from Table 1, the difference between the measured values $\left\{\alpha_{n}\right\}$ for two methods is no more than λ/33. It is also worth mentioning that the error of introducing wavefront aberrations into the laser beam was about λ/20 due to the limitations of the deformable mirror used in the setup. It explains the fluctuations in the measured values in the range 1.5λ ± 0.05λ.

## 4. Conclusions

A theoretical and experimental study of a compact wavefront sensor based on highly efficient computer-generated Fourier holograms was presented. A modification of the scheme developed by the authors earlier was proposed, which allowed one to put the new approach into practice. A comparative analysis of the correlation peaks shape for various wavefront aberrations in the registration plane of a digital camera was carried out in the presence and absence of a Fourier transform lens in the setup. It was experimentally shown that the value of $\Delta \left\{\alpha_{n}\right\}$ between the two methods was no more than λ/33.

## Figures and Tables

**Table 1:** The result of wavefront aberrations measurement for two different implementations.

Focal Length	Polynomial Type	$\mathrm{Value}\;\mathrm{of}\;\left\{\alpha_{n}\right\},\;\mu m$		$\Delta \left\{\alpha_{n}\right\}$
		Fourier Lens	Digital Fresnel Lens	
250 mm	$Z_{2}^{2}$	1.52	1.50	λ/50
	$Z_{3}^{-1}$	1.49	1.52	λ/33
500 mm	$Z_{2}^{2}$	1.50	1.53	λ/33
	$Z_{3}^{-1}$	1.52	1.53	λ/100
1000 mm	$Z_{2}^{2}$	1.49	1.49	λ/100
	$Z_{3}^{-1}$	1.54	1.52	λ/50
